# Religion, Kinship and Health Behaviors of African American Women

**DOI:** 10.1007/s10943-013-9784-0

**Published:** 2013-10-19

**Authors:** Kathryn Coe, Colleen Keller, Jenelle R. Walker

**Affiliations:** 1 Department of Public Health, Indiana University-Purdue University Indianapolis, Indianapolis, IN USA; 2 College of Nursing and Health Innovation, Arizona State University, 500 N. 3rd St., Phoenix, AZ 85004-0698 USA

**Keywords:** Religion/spirituality, Relationships/research, Obesity/overweight

## Abstract

A positive relationship exists between functional health and religion. We present an empirical definition of religion and describe the key elements of religious behavior, building a model that can be used to explore the presumed relationship between religion and health. Semi-structured interactive interviews were conducted with 22 participants over a 6-month period. Head Start programs and churches located in the inner city of a large metropolitan area. Twenty-two African American women were aged from 21 to 45. We focus on social relationships and propose that prophet-created religions mimic kinship relationships and encourage kinship-like cooperation between members.

## Introduction

As a significant number of researchers have conducted studies indicating that a positive relationship does exist between functional health and religion, there has been a proliferation of such studies over the last decades (Arcury et al. 2007; Benjamins 2004; Ellison and Levn 1998; Koenig et al. 1999; Koenig 2001; Pargament 1997; Pargament et al. 1998). As an example, a review of faith-based physical activity interventions that were targeted toward African Americans (Bopp et al. 2012) indicates that significant improvements were observed in indices related to cardiovascular disease such as blood pressure, energy expenditure, physical activity and dietary habits, and body composition variables were obtained (i.e., weight and BMI) (Baruth et al. 2011a, b; Bopp et al. 2012; Wilcox et al. 2007, 2010). In regard to compliance or participation, faith-based studies indicate that pastoral support, which is modeled on the paternal role, is associated with higher rates of follow-up participation for physical activity (Baruth et al. 2008).

While the results of these studies are intriguing, a number of criticisms have been leveled at them related, ultimately, to the fact that religion is considered to be a multidimensional or multifaceted phenomenon. Controlling all the independent variables is problematic (Ferraro and Albrecht-Jensen 1991; Kirkpatrick 2006). It is not clear what mechanisms might underlie the connection between the independent variable, religion, and the dependent variables (e.g., increases in social support, discouragement of certain risk behaviors and psychological effects related to the performance of rituals). A related problem, however, is that we have not defined religion, other than to provide a laundry list of what it may involve (e.g., beliefs, feelings, attendance, rituals, feelings and supernatural beings).

One key universal characteristic of religion, drawn from a large number of studies, is the use of kin terms in religious contexts, as, for example, a priest may be called father while a nun may be addressed as mother or sister. Members of congregations may refer to one another as children of God, the Father, and as brothers and sisters to one another. The widespread use of these kin terms in situations that do not involve biological kin (e.g., religions, sororities and fraternities, tribes and clans) seems to have a common effect: it helps create a metaphorical family, the members of which tend to act as kin, preferentially cooperating with and sacrificing for their metaphorical relatives. While religion is not the same as a sorority, as the cooperation may be much more intense, this particular characteristic of religion is ancient and found around the world in all societies (Steadman and Palmer 1995).

In this paper, we argue that a focus on the kinship aspects of religion will help throw light on the role that religion seems to play in health behaviors. We test these assumptions in a population of young African American women at risk of cardiovascular disease.

## Theoretical Rationale

In this paper, we use an evolutionary, reductionist and empirical approach and borrow from Steadman and Palmer (Steadman and Palmer 1995) who define it in terms of behavior, namely they see religion as a form of communication involving the making and acceptance of supernatural claims, one of which is that all members of the religious group are actual and biological who share a supernatural father. While religion may be much more than this, what this definition allows us to do is begin to examine the effects of the claim that all members are actual kin. What Steadman and Palmer argue is that one effect is a growing development of kinship-like cooperation. Religion, they argue, appeared very early in human evolution, a time when humans lived in small groups of kin, practiced traditions that were honed through time, and honored their ancestors, who were said to be the first ancestors, the ancestors shared by everyone in the religion, and the individuals who were the source of traditions, including those related to the existing kinship structure and the behaviors expected of those who had specific kinship ties. Supernatural claims regularly associated with religion include the claim that as members of a congregation share a supernatural father, they are kin and expected to behave as such. In religions, as in families, brothers and sisters are encouraged to behave altruistically toward one another. Further, the shared metaphorical ancestors (e.g., a supernatural being who is father or perhaps a mother, saints) are used to provide authority to rules of social behavior and its enforcement. When we are involved in a church, we accept that authority. When two individuals who identify one another as kin also cooperate reciprocally during rituals and other religion-associated activities, the social relationship is further strengthened.

This discussion suggests that part of the importance of religious involvement may lie not in “unspecified psychosocial influence” but rather in the close, trusting and enduring social ties—kinship-like relationships—regularly encouraged by religions (Levin and Vanderpool 1989). Research indicates that not only do religions regularly encourage social ties (Koenig 2001), but that social ties and the lack of such ties are related to health and its absence.

Social support is defined as the extent and conditions under which the context of interpersonal ties and different types of support might be provided by people holding different roles (e.g., a mother versus a sister, biological or metaphorical). These roles, in turn, are associated with certain social obligations and linked to determinants of well-being, and that “social support” always describes positive, and giving, aspects of social relationships. In this paper, we describe the key elements of kinship ties and point how they are found in churches. We then focus on describing African American women and their church relationships. African American women who are at increased risk of morbidity and mortality from cardiovascular disease (CVD) compared with White women (Go et al. 2013; Sudano and Baker 2006).

## Key Elements of Kinship and Social Support

Kinship, or close enduring social relationships, according to a number of scholars, was of primary importance to our distant ancestors (Lancaster and Whitten 1990; Lee and DeVore 1968). The human (and mammalian) strategy of altricial birth and prolonged immaturity of offspring necessitates intense, long-term maternal (and possibly paternal) care. All parenting, or bringing in others, generally kin, to help protect, nurture and teach one’s children also seems to have been an important human strategy (Hrdy 1999). Humans seem to possess a wide range of proximate mechanisms that enhance the probability of their forming nurturant relationships (Silk 1990) and maintaining these nurturant relationships through time.

The core of kinship is the parent–child relationship. The parental obligation is to provision, protect, nurture and guide, or educate the child until that child reaches mental, emotional and physical maturity (Shepherd 1980). The child, to learn the skills necessary for survival, was designed to be influenced by those acting in such a hierarchical role. Out of the relationship between parent and child, and identified by it, stems relationships with other kin, including ever more distant kin (e.g., aunt and uncles, cousins, second cousins). This suggests that humans may have been designed to learn best from kin or from individuals who interact with them as if they were performing a kinship-like role, particularly a parental one. As learning also appears to be age-specific, with different tasks learned at different ages in the life span, the transmission of behaviors may be an on-going process, with much learning occurring in the family during childhood (Halfon and Hochstein 2002). A number of lines of evidence appear to point to the fact that these kinship relationships form the models we use to form relationship outside of kinship.

Religions replicate the kinship model in that they regularly use kinship terms metaphorically (e.g., “mother,” “father,” “sister” and “brother”) to refer to supernatural beings (God the Father), the religious hierarchy (the roots of the word “pope” comes from the Greek pappas, meaning father) and members of the congregation. Further, religions regularly encourage kinship-like cooperation between family members these metaphorical kin (Steadman and Palmer 2008). In addition, religions regularly encourage respect for elders and ancestors—the church fathers—and encourage good parenting. Further, both kinship systems and prophet-created religions are hierarchical with an asymmetrical transmission of knowledge, which is a responsibility of the elders who serve as educators involved in the transmission of knowledge to the young and the encouragement of certain behaviors throughout the life span.

Available evidence appears to support that health behaviors (negative or positive) are often learned from kin; to say that the etiology of diseases such as CVD is “partially related to family environment” (Venters 1989) is to implicate behaviors (negative and positive) learned in the home. Evidence supports that the early stages of CVD begin in childhood and adolescence (Venters 1989; Voors et al. 1977) at which time of life, socialization by family into certain negative practices of eating and physical activity, largely through example, is occurring (Voors et al. 1977). Socialization also seems to be linked to positive health practices such as seat belt use, exercise, nutrition, medical and dental care and screening examinations (Higgins and Dicharry 1991; Venters 1989; Voors et al. 1977). Social bonding is associated with health (Troyer 1988), in that adults with strong supportive relationships appear to be better able to cope with stresses in the environment (Kahn and Antonucci 1980). In addition, the most appropriate format for teaching health behaviors may indeed be parental, in the sense that it involves personal, or one-on-one teaching (Hall et al. 1977), or family-oriented teaching (Venters 1989). Particularly important in personal teaching may be modeling and guided practice (Marty et al. 1983). Edwards (1980), for example, found that modeling alone may be as effective as combinations of other methods to teach health behaviors and to change negative ones.

In sum, this study assumes that the following were characteristics of our ancestors (and our) enduring kinship social relationships and religions:

1. The mammalian strategy of maternal care suggests that parents and offspring are tied into a relationship of interdependency and that close, enduring social relationships may be necessary not only to human well-being, but for survival. The differential investment of each sex in offspring suggests that each may demonstrate a distinctive, yet complementary caretaking behavior. These behaviors are reflected in church hierarchies and the behaviors they encourage.
2. Altricial birth and prolonged immaturity of human offspring suggest that, on average, maternal (and perhaps paternal) care will be long term. The identification of individuals as kin is a parental strategy. These teacher–student relationships would have been hierarchical; however, one factor necessary for the transmission of knowledge may have been mutual influence, with both participants open to the influence of the other. Behaviors often would be transmitted through observation and modeling, and behaviors learned in this manner may be difficult to modify, not only because they may be tied into inextricable from a complex web of enduring social relationships and important and remembered social events, but also because human children perhaps have been selected to learn from individuals acting in a specific type of relationship (i.e., hierarchical) and to retain that knowledge. Churches frequently talk about witnessing, which refers in many instances, to providing appropriate role models.
3. The theory of kin selection suggests that kin will be likely to intervene if adolescent or adult behavior is seen as culturally or biologically maladaptive. Also, humans may be more likely to cooperate with sacrifice for close kin (or individuals who behave as close kin) than they are for distant kin or non-kin. For example, a mother may be likely to sacrifice her own pleasures (e.g., smoking) to protect offspring or to ensure she is around to see them mature. Kin also may be likely to share time and scarce resources with close kin. This cooperative and sacrifice, given the human propensity for observational learning (modeling and copying) (Bandura 1986), may trigger reciprocal actions. In addition, individuals may be more likely to affected by the illness and death of close kin, or individual with whom they share a close enduring social relationship, and thus more likely to modify associated behaviors. The close monitoring of behavior and guiding behavior is reflected in religious organizations. Further, churches frequently are a center of kinship, conducting important rituals related to birth, death and even puberty.

## The Measure of Religion

Members of religious organizations and researchers frequently claim that faith is a necessary element of religion. Faith is said to drive religion actions, just as other human behaviors commonly are said to be motivated from within, or caused by, various needs, impulses, instincts and beliefs (Bandura 1986). Thus, faith has come to be the definition and measure of religion. Although faith may be an important element of religion, such formulations are problematic. First, such arguments often are tautological; inner determinants often are inferred from the very behaviors they supposedly caused (Bandura 1986). In addition, internal causes are difficult to ascertain (individuals are not always privy to the factors governing their behavior (Bandura 1986). Also such formulations are not easily testable or refutable by empirical evidence (Bandura 1986; Palmer et al. 2008); whatever else modern prophet-created religions may do (Durkheim 1954; Eng et al. 1985), they regularly seem to involve the use of kinship terms in a metaphorical way to refer to non-kin. Not only are kin terms used to refer to unrelated individuals in the church, they are used to refer to supernatural beings. When all members of a church claim that they share a common ancestor, they are implying that they are members of one family. Their use of sibling terms (“brother” and “sister”) supports this claim. As anthropologists have noted, the use of such terms encourages certain behaviors between those using the kin terms (Kroeber 1909). For example, sibling-like cooperation is expected between individuals who refer to each other as “brother” or “sister.” Parental terms (“father” and “mother”) seem to carry with them a number of obligations, including, it can be argued, an educational responsibility.

The focus of this exploratory study is on close, enduring social relationships, kinship or metaphorical kinship, and their relationship to religion and their effects on health behavior. Specifically, the aim of the study is to describe the effect of such social relationships, as found in either biological or metaphorical kinship (i.e., in religion), on health behaviors related to the incidence of CVD. The population for this study consisted of young African American women, at risk of CVD, who resided in a large metropolitan area in the southwestern USA.

## Materials and Methods

### Design

Kinship systems and religions may share the following characteristics, which form predictions for this study. The research questions that directed this study were: (a) How do members of religions groups use kinship terms to refer to fellow members of the group who are not literally kin? (b) Although religious conversion may disrupt relationships between biological kin (when family members, who are not members of a new church or cult, disapprove), how are church members encouraged to establish and maintain long-term or enduring family-like ties with other church members? In older, established churches with multigenerational membership, close enduring cooperation may be encouraged in both biological and metaphorical families. This cooperation may involve sacrifices of time, resources and ego. Finally, (c) How does the church serve as educational body? Adults, both kin and metaphorical kin, perform an educational role, often teaching through modeling. There will be behavioral differences between the sexes in this educational involvement. Respect for the elders may be an important part of the transmission of knowledge, and (d) How do metaphorical kin, along with kin, intervene if the behavior of young (biological and/or metaphorical) kin is seen as maladaptive?

Using naturalistic inquiry, a qualitative descriptive design with semi-structured interviewing was used to gather data from a sample of African American women. Conventional content analysis was conducted to identify culturally specific views of African American women concerning the processes of kin, metaphorical kin and religion to health and health behavior.

### Sample

A convenience sample of 22 African American women, aged 21–45, was recruited for this study through Head Start programs and churches located in the inner city of a large metropolitan area in the southwestern USA. Women were included if they read and understood English. Participants, who responded to recruitment from administrators at the Head Start center and announcements in church bulletins, were both members and non-members of churches. A theoretical sample was selected to ensure that a wide range of incomes, occupations, education levels, marital status and family sizes were represented. This study was part of a large study of 80 African American women that included a health assessment and an examination of risk factor status. This study was approved by Institutional Review Board of Arizona State University.

The potential participants were given a contact release form to sign. The form was read for those participants who were unable to read or write. The investigator contacted the participants and met them on a convenient date, time and place. Following the assessment of demographic data, and if they met the inclusion criteria, an information letter that included a summary of the study’s purpose, procedure and role of the participants was read to each participant.

### Interviews

Demographic information was collected, and semi-structured interactive interviews were conducted with 22 participants, after their health assessment, over a six-month period. Each subject was interviewed one time, for approximately 2 h, and re-contacted to confirm the coded findings following data analysis. Semi-structured interviewing was used to concentrate and narrow the focus to young African American women’s own perspective and viewpoint related to health, support for healthy behaviors and the role of religious beliefs in health. All recorded interview data were transcribed verbatim immediately postinterview and then analyzed using conventional content analysis methods (Krippendorff 1980; Miles and Huberman 1994; Weber 1990). The interview began with the open-ended question “What is health?” All interviews were audiotaped and transcribed verbatim. Data were subjected to content analysis, and categories were quantified. Data also were collected in informal interviews with pastors and lay leaders of the churches.

The two primary investigators independently read each data transcript from beginning to end, then, sentence by sentence and line by line manually highlighting the culturally specific views of causes and processes health and religion relationships, using key words. These key words were used to create codes after reading three-to-four transcripts. Then, these codes were used to label the remaining transcripts. New codes were added if data did not fit into the preliminary codes. After coding, all data within each code were read, similar codes were merged and broad codes were divided. Conceptual themes were developed from the data to address the conceptual questions guiding the open-ended interviews. Means, frequencies and percentages were calculated for the demographic data, obtained at the time of enrollment.

The trustworthiness and authenticity of the process of data collection, data entry, transcription, analysis and interpretation were described in detail so that an audit trail was created (Miles and Huberman 1994). After transcribing data obtained from the interview, each participant was asked to review the transcripts produced by the investigator for accuracy, and if the participant was unable to read, the investigator read the transcripts to the participant. Specific participant quotations are deployed in the text below, using pseudonyms for participant names.

## Results

### Sample Characteristics

The 22 women who participated in this research were aged between 21 and 40. Fifteen women were employed; 13 worked in clerical and secretarial positions, 1 was a social worker and 1 was a state health education administrator. Seven of the women had education levels less than 12th grade; 9 were high school graduates; 4 were college graduates; and 3 had postgraduate education. Eight of the participants were married. Eleven of the women had children, for a group total of 28 children. Four of the mothers were married; seven were not.

Twenty of the 22 women were at risk of CVD, either because of family history, overweight, or lack of exercise. Sixteen of the participants had a history of CVD in their families; nine of these had experienced death of kin, and two had experienced the near loss of a mother to heart attack or stroke. Twelve of the participants, using current health standards, were overweight, 6 women were from 10 to 20 pounds overweight, 5 were 20–30 pounds overweight and 2 were 50–60 pounds overweight. Although all participants reported that they exercised albeit on an irregular basis, five reported they exercised on a regular basis. Only three of the participants reported that they presently smoked.

### Kinship, Religion and Health

Twenty participants, when asked about the three most important things in their lives, mentioned kinship (family, children, parents or husband). Seventeen of the 22 participants in this study specifically mentioned that religion was important. Nine claimed that God or the church was the most important things in their lives. Eight of the 17 participants either placed religion as the second most important thing in their lives or they mentioned that religion and/or church were very important. Not only were religion and kinship important factors in the participant’s lives, but the two frequently were associated and interdependent. For example, one participant stated: “God is number 1, then my mom and dad.” Another said: “God is first, then my marriage,” and “God is the most important thing in my life. If I put him first in my life, then everything else will fall into place: my marriage, my family and my life.”

Three of the 22 participants did not mention religion at all in their interviews, and one subject claimed she was the “black sheep” of the family because of her lack of interest in the church. This participant dismissed religion, claiming that: “Too many people feel that God will put them through and they don’t do anything for themselves… prayer is fine, but you don’t just pray and sit back and wait till things happen.” Another one of the three participants mentioned religion only to say that the man in her life was: “An all right person, he’s into religion.” The third participant, who claimed: “I’m not into church right today, believe me! You want to stay health because God want you to stay healthy. Being raised in the church, you can be the richest person on earth and not have a dollar.”

#### How do Members of Religions Groups Use Kinship Terms to Refer to Fellow Members of the Group Who are Not Literally Kin?

The social organization of the churches to which participants belonged was family-liked, in the sense that members used kinship terms for each other. Members, as the examples will show, referred to each other as “brother” and “sister” and to older men and women as “mother” and “father” and “godmother.” As participants didn’t distinguish between metaphorical and literal kin, it was difficult at time to separate the two, unless the interviewer probed for more information.

The pastor, who was treated as the father of the metaphorical family (in the sense that he was treated differently and he demonstrated an interest in his children and involved himself in their problems and celebrations), had specific educational responsibilities, instructing members about their obligations to one another. This encouragement may be an important factor in influencing behaviors (Castro et al. 1991). He was responsible for maintaining harmony in the church. He demonstrated an interest in his “children,” and he was called in settle their disagreements and participate in both their grief and joy. Six members of the church mentioned going to him with their problems and using him to settle disagreements. For example: “If it [a problem] is serious, I probably go to my pastor,” and “[When I get angry or down] I might try to catch Rev. Smith.”

Although one subject, who was high on drugs during the interview and quite angry with men (particularly the fathers of her three children), voiced her dissatisfaction with the pastor’s involvement in her problems, the pastor felt he was trying to guide her choices in ways he felt were appropriate.

I told him one time the he ain’t nothing but a damned educated fool. He says, “Charlayne you shouldn’t tell me things like that, but it’s true.” He says, “I’ll be right over to see you. How are your feet? How’s your back? If you try to come on more Sundays, maybe your feet wouldn’t hurt.” Then he wants to yak, yak, yak, and I am not in the mood to hear that today.

#### How are Church Members Encouraged to Establish and Maintain Long Term or Enduring Family-Like Ties with Other Church Members?

Several lines of evident support that enduring ties were encouraged. First, participants were regularly encouraged to get involved in the church activities and to maintain social ties to the church throughout their lives. As one participant related, “I was raised to go to church.” It was an every Sunday ritual. Mom and dad taught [me] the values that it was wrong to leave the church. You suffer from it.

From the time I was a little girl, faith and belief in God was the most intricate part of my family structure. I went to church every Sunday, Sunday school, vacation Bible School and all that kind of stuff. It has just helped me to become a better person.

Mothers played the strongest role in encouraging this enduring commitment to the church:

My mother always stressed towards religion. My father he never stressed any religion to us. My mother is a church-goer. She always stressed to start going to Sunday school and going to church and all. She said as time goes by you will get into the habit of just getting up on Sunday mornings and going to church.

I send my three sons to church ‘cause there are some things you can learn there that you may not learn just being on the streets and you need that higher authority to make it through each day.

Parental involvement with the church led to the involvement of offspring in the church. Children were drawn into church activities along with their parents, and children became active in the social relationship maintained by the parents: “I watched my mother a lot when I was little, ‘cause she’s a church lady. Always in the church, and uh, she loved to sing in the choir, and I love to sing. I took my mother as a role model.”

All of the participants had inherited their religion from close kin, generally their parents, who had, in turn, inherited from their parents. Participants, in turn, transmitted religion to their children. Thus, the enduring ties lasted only a number of years, they lasted a number of generations: “I came from a Christian family and a Christian home and we were disciplined and the Bible says train up a child the way that he should go, and when he is old, he shall not depart from it. That is what happened. I have not parted from it,” and “When I was growing up, it [church] was very important…and now my three sons they have to go to church.”

#### How Does the Church Serve as Educational Body?

In a number of cases (*n* = 7), participants mentioned that the church was educational. The church educated individuals in regard to their social obligations to each other and, in some cases, taught and/or reinforced behaviors seen as healthy: “In some people’s religion, you can’t smoke. So if you in a church and you can’t smoke, well then, you gonna quit smoking cigarettes.”

When I was about 16 or 17, I think I started realizing that my body is a temple of God and that I shouldn’t put unnecessary things into my body because if I do, I will be harming my body. While I was in high school I never did drugs or smoked pot or anything, even to this day, I don’t touch that stuff. Even though I was influenced because I was around kids who did that and they would call me names and call me square and say that I did not fit in with the crowd and stuff. I didn’t do it because of my relationship with God, plus my mother. She always stressed not to do drugs.

Mothers, who were the ones most likely to encourage certain behaviors including health behaviors, often used the teachings of the church to reinforce what they taught: “When I was growing up, my mother would say to us to always try to eat healthy foods, fruits and vegetables. Try to eat a well-balanced meal and to exercise.”

My mom was probably the biggest influence. I saw her bring up 13 kids practically alone. She had her family to support her, but she did the biggest amount of work. I can truthfully say that all of my brothers and sisters are in excellent health and they haven’t been in any trouble. If we did something wrong we were reprimanded. We did not get away with it. She wasn’t very strict, but you knew that she was in control.

[To keep my kids from doing drugs and alcohol] we sit down and have a family conversation. When we get up in the morning, we start with prayer, we will have a devotion prayer and in the evening we sit down discuss things that went on during the day.

Also important in the educational aspects of the church were relationships between young women and older women. Five participants claimed older women in the church held important influence: “When I have a problem, I talk to older women like my godmother. Of the people I look up to, I admire their kindness, the religion part of them, the way they are always trying to help you,” and “My husband teases me because I have a friend who is 75 and one that is 65. He said that I have to be around old people. It is not that. It is just that I enjoy the differences in ages and what they can tell me and I guess that they have an influence on me.”

#### How do Metaphorical Kin, Along With Kin, Intervene if the Behavior of Young (Biological and/or Metaphorical) Kin is seen as Maladaptive?

Kin and metaphorical kin actively discourage behaviors seen as negative: “When I wrote the bad checks, she [mother] was hurt. She cussed me out and asked me what was I on? She asked me if I regretted what I did. She was like at her last straw… girl don’t screw up this time,” and “My mother keeps telling me that you got to lose weight because high blood pressure runs in the family.”

Negative behaviors were not only criticized, but also punished: “I sent for my sister twice and I had to send her back because I didn’t like her habits. No you cannot bring boozers into the house. [I told her] I don’t set that example so you have to go back home.”

Further, kin rewarded individuals who were seen as attempting to change negative behavior:

When I go out of jail I went to church. First time I ever listened to the music I just cried and it was not that the music was good it was just that I was hearing the music. I was just sitting in my pew, I just couldn’t stop crying. I kept wiping my face and they [members] saw me and they passed some Kleenex to me and it was like, girl, that is a good sign that you cry in church. After church I talked to the pastor and his wife and she said a prayer for all of us. I was crying again.

Participants appeared to feel that it was important for others to monitor their behavior and remain involved in their lives. One woman claimed that when she left the church for “maybe a month or two,” she lost her paycheck, gained weight and fought with her kids. When she rejoined the church, these behaviors stopped.

The church, according to fifteen participants, encouraged behaviors such as sharing, kindness, helpfulness and goodness. These were the same behaviors that mothers encouraged between offspring: “I am from a religious background. We were just always taught the commandments, just generally to treat each other fairly and that is the only thing that I ask or require from others,” and “When I was growing up, church taught me sharing, you know, don’t do unto others as they do unto you. Be better to people than they are to you.”

Helping or cooperative behaviors were not only encouraged, but also expected and delivered. Members claimed that they frequently helped others and that they enjoyed doing so:

“I know I want to be there for people if they need a shoulder to cry on,” and, “I stayed there and watcher her [my godmother] and helped her cook, fed her, whatever needed to be done…When [my aunt] got sick, I had to end up stepping in. I stayed there day and night. I did that for about six months or more.” “My sister and I took turns taking care of our godmother. We made her sit down, made her eat the right foods. We cooked the food and took it down to her. She did fine.”

Not only did participants expect to help others when they needed help, they felt that they could expect help when they needed it. Support was reciprocal: “When I was in jail, one of my sisters from the church took care of my babies,” and “When I was shooting drugs, I was a smart thinker because I took my son home to my mother, where I knew that he would get what he needed and vegetables, shots, and schooling and everything.”

A number of participants mentioned that they had given up negative behaviors in order to protect kin in a variety of ways. Two participants, for example, mentioned that they had stopped negative behaviors to protect parents, particularly mothers, who would be hurt by possible negative consequences:

I stopped coke for my mom because for a while she did not know that I was doing that and she is a senior citizen now. I don’t want to have her dealing with my 13 year old and a 5 year old; and for my kids because I was not spending any time with my kids at all and they know something was wrong. I prayed when I was in jail. I had to do some real soul searching. It is time to think about my mother now, because she was always there for me. I straightened up because my mother is getting another divorce and I want to be there for her. She would have to take care of the kids and I didn’t want to cause her no heart attack on my mother. It is time to think about my mother now, not only of myself, because she has always been there for me, I never had to want for nothing, nothing.

Three participants claimed that they had changed negative behavior because of the possible effects of their children and grandchildren: “If I die and leave [my kids], who is going to give them the right nutrition, you know who’s going to take care of them and the only way I can do that is to change myself, set an example for the kids to eat,” and “I behave because I want my children to see me live to be an old lady and I want to see my children grow up to have children.”

Participants also claimed that they attempted to changed negative behaviors because of a responsibility to others in the church. They felt that they would influence people by their behavior, negative or positive. In other words, they were conscious, and they were role models:

Putting others first is probably from the Christian ethic of helping and doing things for other people and if I don’t take care of myself, I’m not going to be here to do anything for anybody else.

I think about what I want the younger generation to learn ‘cause I feel that if I do it myself, then I can set a good example for the younger generation. Maybe they can come to me with a problem. But if I’m out there doing the same thing you’re doing, I can’t help you. I want to be a role model for somebody that may not have a role model. So I figure the only way I can do this is to keep in shape, eat the right foods, and stuff. I can’t very well sit at the table and tell people to ear carrots when I don’t like carrots.

## Discussion and Conclusion

In sum, the predictions regarding the similarities between kinship and religion were supported by the evidence collected in this pilot study. Further, in this study, as in others, kin (metaphorical or literal) were a primary source of social support (emotional, informational and economic), and this social support was related to health (negatively or positively). Kin and members of the churches, who were organized in a kinship manner, influenced the health of individuals by teaching health behaviors to the young, by encouraging behaviors seen as healthy and by attempting to modify behaviors seen as maladaptive. This transmission of knowledge was facilitated by the encouragement of the maintenance of close family ties and respect for the elders (the pastor or older women) and the knowledge they possessed.

Although some of the behavior encouraged by kin was healthful (by current standards), some was not. Not all of the behaviors encouraged by kin (metaphorical and/or literal) fell into the category of what health providers regard as appropriate in health promotion. The argument contained in this paper is not that the health behavior encouraged by kin will be identical to health behaviors encouraged by health providers. Clearly that is not true. In two cases, parents encouraged daughters to gain weight, even though such a gain would have put them into the overweight category. The argument is that health behaviors are encouraged in such relationships and that behaviors learned in such a way may be difficult to change. What is important to this paper, however, is the fact that knowledge was transmitted between individuals involved in enduring relationships and the fact that behaviors maintained by the women in this sample—behaviors they found difficult to change—was learned from individuals who were literal or metaphorical kin.

All the women interviewed in this study indicated that they did have an understanding of factors, such as diet and exercise, which health providers and teachers had taught them were important in health. Twenty of the women were aware of a relationship between behavior and CVD. However, knowledge clearly was commensurate with behavior, except perhaps on a temporary basis (a diet that was not continued, an exercise regimen that fell by the wayside). The majority of the 22 women involved in this study (*n* = 19) maintained diet and exercise patterns they had learned in childhood, whether or not those behaviors contradicted what they later learned in school or from health providers. For six of these women, behaviors learned in children were similar to health behaviors learned in school and from health providers. Five of these women were raised on farms where fruits and vegetables formed the bulk of their diets; one had a parent employed in purchasing produce. Three of the women involved in this study did change their health behaviors from negative ones learned in childhood to positive ones. Two of these women changed dietary habits due to death of their mother and other close kin and their close involvement with suffering prior to death. One woman changed dietary habits primarily because of lessons learned in school. However, it may be important to recognize that her dietary changes involved not only a rejection of family knowledge, but also a breakdown in family relationships due to that skepticism. This resulted in the loss of an important social support system.

An important take-away message these data show is the importance of the role the church and kin hold in the development of intervention strategies in African American women. Clearly, the traditions passed between generations that translate into mutable health promotion behavior, such as valued food rituals, such as intake patterns of selection and preparation can be used as intervention leverage points. The most obvious conclusion which can be drawn from this study is the need for capitalizing on the connection between religion, close enduring social relationships and health behaviors.

While the link between religion and health has been investigated and a relationship appears to be present, the mechanism(s) by which this link operates is unclear. This paper has proposed and provided evidence that religion: (a) replicates kinship, (b) operates to encourage the maintenance of close relationships and (c) is an important vehicle for education of young and the encouragement of certain behaviors among all age groups. If these hold true, churches may be especially appropriate for health education to occur. In this paper, we argue that social relationships, based on kinship, have long played a critical role in the human’s ability to survive and rear offspring in often hostile environments and that the model for and responses to such relationship are wired into our evolved brain and endocrine system. Humans evolved in small groups of close kin; cooperation among those kin, including cooperation in protecting one another, in providing for one another, and in the transmission of knowledge from one generation to the next, would have been essential.
